# Fossils, fish and tropical forests: prehistoric human adaptations on the island frontiers of Oceania

**DOI:** 10.1098/rstb.2020.0495

**Published:** 2022-04-25

**Authors:** Patrick Roberts, Katerina Douka, Monica Tromp, Stuart Bedford, Stuart Hawkins, Laurie Bouffandeau, Jana Ilgner, Mary Lucas, Sara Marzo, Rebecca Hamilton, Wallace Ambrose, David Bulbeck, Sindy Luu, Richard Shing, Chris Gosden, Glenn Summerhayes, Matthew Spriggs

**Affiliations:** ^1^ Department of Archaeology, Max Planck Institute for the Science of Human History, Germany; ^2^ Department of Linguistic and Cultural Evolution, Max Planck Institute for the Science of Human History, Germany; ^3^ School of Social Science, The University of Queensland, Brisbane, Australia; ^4^ Department of Evolutionary Anthropology, University of Vienna, Vienna, Austria; ^5^ Southern Pacific Archaeological Research, Archaeology Programme, University of Otago, Dunedin, New Zealand; ^6^ Department of Anatomy, University of Otago, Dunedin, New Zealand; ^7^ Archaeology Programme, University of Otago, Dunedin, New Zealand; ^8^ College of Asia and the Pacific, The Australian National University, Canberra, Australia; ^9^ School of Archaeology and Anthropology, College of Arts and Social Sciences, The Australian National University, Canberra, Australia; ^10^ UMR 7209 AASPE, CNRS, Muséum National d'Histoire Naturelle, Paris, France; ^11^ CIRAP, Université de la Polynésie française, Tahiti, French Polynesia; ^12^ Vanuatu Cultural Centre, Port Vila, Vanuatu; ^13^ School of Archaeology, University of Oxford, Oxford, UK

**Keywords:** stable isotope analysis, Near and Remote Oceania, Pleistocene, human dispersals, palaeoecology, tropics

## Abstract

Oceania is a key region for studying human dispersals, adaptations and interactions with other hominin populations. Although archaeological evidence now reveals occupation of the region by approximately 65–45 000 years ago, its human fossil record, which has the best potential to provide direct insights into ecological adaptations and population relationships, has remained much more elusive. Here, we apply radiocarbon dating and stable isotope approaches to the earliest human remains so far excavated on the islands of Near and Remote Oceania to explore the chronology and diets of the first preserved human individuals to step across these Pacific frontiers. We demonstrate that the oldest human (or indeed hominin) fossil outside of the mainland New Guinea-Aru area dates to approximately 11 800 years ago. Furthermore, although these early sea-faring populations have been associated with a specialized coastal adaptation, we show that Late Pleistocene–Holocene humans living on islands in the Bismarck Archipelago and in Vanuatu display a persistent reliance on interior tropical forest resources. We argue that local tropical habitats, rather than purely coasts or, later, arriving domesticates, should be emphasized in discussions of human diets and cultural practices from the onset of our species' arrival in this part of the world.

This article is part of the theme issue ‘Tropical forests in the deep human past’.

## 1. Introduction

The cultural and ecological adaptations that enabled our species to expand outside Africa to almost every continent during the Late Pleistocene (125–12 ka) remain major subjects of interest to archaeological, anthropological and biomolecular research [1,2]. Oceania has become an area of particular focus in this regard [3–7]. The earliest human arrival in New Guinea, the Aru Islands, mainland Australia and Tasmania, which then formed the single continent of Sahul (*ca* 65 ka [5]), has been frequently prominently tied into wider discussions of the timing and number of our species' dispersals beyond Africa [8]. However, significant attention has also focused on the Late Pleistocene human occupation of the islands beyond the Sahul landmass (i.e. the Bismarck Archipelago), as early as *ca* 44 ka at the site of Buang Merabak, and what this might indicate in the context of the early capacities of humans to navigate oceanic routes and adapt to isolated insular settings [9,10]. Similarly, though significantly later, the first human arrival in Remote Oceania (i.e. in Vanuatu) approximately 3000 years ago [11], represented by Lapita-affiliated groups, has been argued to represent an important case study for exploring how humans solved the problems of long-distance marine travel and island lifestyles [12,13]. Understanding the process of human dispersals into Oceania has been given additional global importance by modern genetic and archaeogenetic evidence for our species' interaction with other hominin species (i.e. Denisovans) in this part of the world [14].

In the context of both Late Pleistocene occupation of the Bismarck Archipelago, and later Holocene Lapita culture colonization of Vanuatu, sophisticated maritime adaptations have been seen as integral to human migration [11,15–17], as they have in many models of human island colonization elsewhere around the world [18,19]. Nevertheless, while interior tropical environments have, by contrast, often been perceived as depauperate ‘barriers’, there is growing evidence that *Homo sapiens* adapted to inland tropical habitats in Oceania by as early as 50–40 000 years ago [3,20], as well as the nearby Wallacean islands during the terminal Pleistocene [21–23]. Similarly, while zooarchaeological and isotopic research has demonstrated an important role for marine protein from fish and shellfish at early Lapita sites in the Bismarck Archipelago and Vanuatu [11,24], alongside the use of domesticates such as pigs, chickens, taro and yams [13,25–27], more recent research has also highlighted the ways in which Lapita communities in Near and western Remote Oceania had mixed, locally contingent strategies to using marine and terrestrial resources from their first arrival in different parts of the region [28], that also involved the management of the various tropical forest ecosystems they came across [29,30] and exploitation of native terrestrial fauna such as tortoises and fruit bats [31].

Some of the most effective ways of answering questions relating to human dispersals and adaptations, as well as hominin population interactions, involve the multidisciplinary analysis of human remains. However, although a number of early archaeological records exist in Oceania dating as far back as 65 000 years ago [5], and human fossils are dated to 60–40 000 years in Australia [32–34], the human fossil record in the islands to the east of the Sahul landmass is much more poorly understood. Human remains found at Pamwak, Manus Island have been assumed to represent a long-term resident Pleistocene–Holocene population [35], though the dating of these fossils has previously remained unreported. The chronology of human fossil teeth at the site of Matenkupkum, New Ireland [36], discovered in association with an archaeological sequence extending as far back as 41–39 000 cal years BP [10], is similarly unclear. The earliest human remains from Vanuatu are well dated to approximately 3000 cal years BP at the site of Teouma [11]. Stable isotopic approaches have already been applied to this latter site to explore changing dietary and ecological adaptations of human groups upon arrival, suggesting use of marine and agricultural resources from earliest occupation [24,37,38]. However, these studies have, to date, only been applied to bone collagen which is known to emphasize high protein contributions to the diet (e.g. fish) and underestimate low-protein resources (e.g. tropical plants) [23]. Stable isotopic approaches are also yet to be applied to the individuals from Pamwak and Matenkupkum, despite the promise shown by these methodologies in distinguishing human reliance on marine versus terrestrial resources in other parts of the tropics during the Pleistocene [23,39,40].

**Figure 1.**
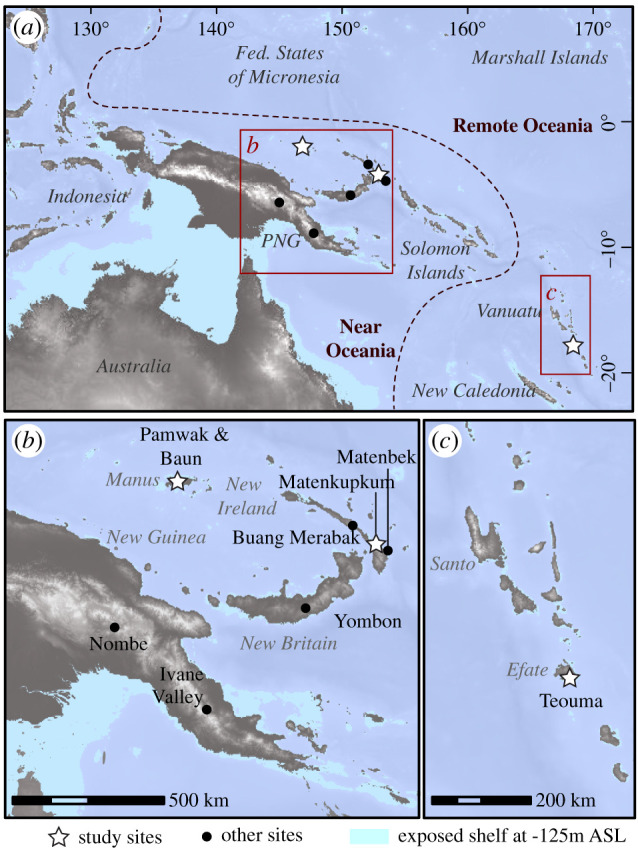
Map showing the studied sites (white stars) and other sites mentioned in the text (black dots), in the wider context of (*a*) Oceania, (*b*) the Bismarck Archipelago and (*c*) Vanuatu.

In this paper, we undertake the first comprehensive chronological analysis of human remains excavated from Pamwak and Matenkupkum, postulated as being the earliest human fossils found in Oceania beyond Sahul (figure 1). We provide a new Bayesian model for the human occupation of Pamwak (including contexts in which human remains were found) and directly date four human teeth found in excavations from Matenkupkum [36]. We also apply stable carbon and oxygen isotope analysis to the tooth enamel of eight individuals from Pamwak, the four dated individuals from Matenkupkum and an individual from Sasi (previously Baun) on Lou Island in Manus [41] (figure 1). We add diverse faunal baselines of terrestrial and marine animals from these sites and analyse microparticles from the dental calculus of five individuals from Matenkupkum, to determine the ecological adaptations practiced by early Oceanian populations in the Pacific realm. Finally, we also apply stable carbon and oxygen isotope analysis to the tooth enamel of 11 individuals, and a marine and terrestrial faunal baseline, from the site of Teouma on Efate Island in Vanuatu (figure 1), in order to complement existing analyses of bone collagen with isotopic data from tooth enamel that are more likely to represent the ‘whole diet’ of an individual [42]. This multidisciplinary analysis of human fossils from Near and Remote Oceania provides an updated picture of the chronology and ecological adaptations of some of the first preserved individuals in a part of the world that is being highlighted in global discussions of human migrations, environmental tolerances and cultural developments and exchange [14,17,43].

## 2. Background

### (a) Human colonization of near and remote Oceania

There is a growing consensus that archaeological evidence for the occupation of the Sahul landmass (New Guinea, the Aru Islands, mainland Australia and Tasmania) approximately 65–50 000 years ago fits with models of early and multiple dispersals of *H. sapiens* beyond Africa during the Late Pleistocene [8]. Genetic analysis of modern Oceanian populations, and archaeogenetic analyses from Teouma and additional sites in Vanuatu, have been used to argue that the earliest groups of our species to reach this part of the tropics interacted with other hominin populations [14]. Within Oceania, movement out from the Sahul landmass into the Bismarck Archipelago is evidenced by around 44–39 000 years ago on the basis of archaeological records at the sites of Matenkupkum and Buang Merabak on New Ireland [10,44], and at Kupona Ni Dari and Yombon in New Britain [45,46]. This early arrival occurred during a time of lower-than-present sea level, though it would still have necessitated significant sea crossings. Manus Island and the Solomon Islands may also have been settled initially during this period, but only a single site from each archipelago is known so far. Although Pamwak on Manus has only been dated back to *ca* 25 000 cal BP, there is a further 1 m of undated cultural deposit to bedrock below this dated context [35,47], suggesting the possibility of a considerably earlier first settlement. Occupation at the site of Kilu on Buka Island in the northern Solomons (Autonomous Region of Bougainville) goes back to *ca* 33 100–31 400 cal BP [48–50]. The sites of Balof 2 and Panakiwuk on New Ireland [51,52] date to approximately 18–16 000 cal BP at a time when cultural connections appear to have intensified between New Guinea and the Bismarck Archipelago. Human activity then seems to increase again around the island region after the Last Glacial Maximum, between approximately 18 000 and 10 000 cal BP [53–55].

Pleistocene colonization of the Bismarck Archipelago and subsequent ‘pulses’ [53,54] or phases [55] of behavioural change have been seen as involving various forms of human adaptation to ‘depauperate’ island settings, with limited faunal opportunities beyond small mammals, birds and reptiles [56]. A perceived lack of animal and plant resources in the interior rainforests of these islands had led to the dominance of a model of resource use whereby small groups of hunter–gatherers mainly focused on reef and coastal resources [57]. Many of the early sites of occupation within the Bismarck Archipelago are coastal, and first arrival would have necessitated an open sea crossing. Moreover, analysis of the shell midden of Matenkupkum shows selective shellfish gathering, requiring close knowledge of the ecological dynamics of the taxa available that may even have been ‘over-predated’ [9]. Early evidence for shellfish exploitation is also observed at Buang Merabak [58]. However, there is little direct evidence for the over-exploitation of marine resources in the Bismarck Archipelago until 20 000 years ago [10]. There is also zooarchaeological evidence for the use of interior rainforest resources at Yombon 25 000 years ago [45,59]. The faunal assemblages at Buang Merabak and Matenkupkum also include forest birds and bats [60], while there is evidence for the exploitation of native rodents and reptiles in the Solomon Islands during the Pleistocene [49]. By 20 000 years ago, the data point to the movement of the northern common cuscus *Phalanger orientalis* between islands, presumably as a source of protein [9] and, later, the introduction of the bandicoot (*Echymipera kalubu*) and spotted cuscus (*Spiloscuscus kraemeri*) to Manus at about 13 000 years ago [47]. Finally, botanical evidence from New Ireland and the Solomon Islands has indicated the use of interior tropical plants, including perhaps some form of arboriculture, as early as the Pleistocene [47,61–63].

During the late Holocene, there was a second major process of human dispersal into Oceania, associated with the expansion of the Lapita Cultural Complex and Austronesian languages [13]. Beginning in Southeast Asia, people carrying distinctive dentate-stamped decorated pottery arrived in the Bismarck Archipelago 3300–3100 years ago, after skirting the northern coast of New Guinea [64] and reached Remote Oceania approximately 3000 years ago [11]. This set of dispersals has been associated with the capacity to undertake long-distance sea crossings, often with no land in sight and a sophisticated ability to extract marine resources. At Teouma, on Efate in Vanuatu, early human populations have been argued to have had a preference for reef and inshore marine fishing, complemented by freshwater resources [65–67] and agriculture in the form of pigs, chickens, yams, taro and bananas, carried from Near Oceania [24,38]. The arrival of the Lapita Cultural Complex has been linked to the idea of ‘transported landscapes', both here and in later settlement in Polynesia [13,68], with significant modifications to island environments occurring as a consequence of agriculture, domesticated animals and commensal species (e.g. rats) [47,69]. However, there is also a growing recognition that Lapita-affiliated populations, and those that followed, likely adapted to their varied new island homes. At Kamgot on the Anir Islands in New Ireland, microbotanical and zooarchaeological research have demonstrated diversity in human diets from first arrival, with a balance maintained between the land and sea and between domesticated, hunted and gathered resources, with mobile societies exploiting various parts of the terrestrial and marine landscape [28]. Similarly, the management and use of tropical forest plants, and the heavy utilization of endemic fauna such as fruit bats and tortoises [31,70], have been reported for Vanuatu [24,30,71] and elsewhere [29].

### (b) Stable isotope analysis and past human adaptations in the tropics

Stable carbon (δ^13^C) and oxygen (δ^18^O) isotope analysis of human and animal tooth enamel has emerged as a key method for exploring past diets and environments in the tropics. In inland contexts, δ^13^C distinctions between C_3_ and C_4_ plants are useful for determining the relative reliance of taxa on C_4_ grassland and C_3_ shrubland, woodland or forest [72,73]. Within tropical forest ecosystems, vegetation growing beneath a closed forest canopy is strongly depleted in ^13^C due to low light [74] and large amounts of respired CO_2_ [75], leading to a ‘canopy effect’ that results in CO_2_, soils, and plant tissues having lower δ^13^C values [75,76]. These δ^13^C distinctions are passed into the tissues of their consumers with subsequent trophic effects of approximately 1‰ [42]. Like terrestrial C_4_ plants, marine plants have higher δ^13^C than all C_3_ terrestrial plants [77,78]. As a result, terrestrial C_3_ and forest-dwelling consumers can be distinguished from marine and C_4_ consumers [79], with more nuanced interpretations possible (e.g. when C_4_ consumers can be ruled out in inland biomes) where detailed faunal baseline references are available. In the case of this study, for later contexts where agricultural resources are available, it is important to note that yams, taro and banana are all C_3_ crops (see also [24,38]).

We expect pre-industrial human enamel bioapatite for individuals relying completely on tropical forest, open C_3_ resources, and marine /C_4_ resources to have δ^13^C values of *ca* -14‰, *ca* -12‰ and *ca* -4–0‰, respectively [40,79]. δ^18^O measurements of animal tooth enamel can provide additional ecological insights into water and food sources. In the tropics, δ^18^O primarily reflects evaporative potential and the source effect of rainfall [80,81]. δ^18^O values have also been argued to distinguish terrestrial from marine consumers [82]. While many isotopic studies in archaeology, including previous studies at Teouma [24], typically focus on bone collagen in order to obtain insights into human and animal trophic levels, it is often poorly preserved over longer time periods. Furthermore, bone collagen δ^13^C is primarily reflective of protein contributions to the diet. The bioapatite structure of tooth enamel is not only more resistant to post-mortem diagenesis [83], but will also record δ^13^C values representative of the ‘whole diet’ during the period of enamel formation, which will vary depending on species and tooth sampled [84]. δ^13^C and δ^18^O of human tooth enamel have recently been used to provide novel insights into varying human reliance on forest and coastal resources in different parts of tropical Asia [39,40], including Wallacea [23].

## 3. Methods

### (a) Radiocarbon dating

Radiocarbon dating is by far the most precise and widely used absolute dating method for organic material. One of the prerequisites of the method for the production of accurate results is sufficient decontamination of the dated material from exogenous carbon found amply in depositional environments in the tropics. In the case of teeth, while both enamel and collagen extracted from the dentine can be dated, the most dependable results are produced by the latter.

We selected four human teeth from Matenkupkum for dating. The Matenkupkum teeth (MAT 001, 007, 008, 011) were found by Chris Gosden and his team in 1988 in Spits 4 and 5 Square B excavated at the back of the cave and away from the main sequence (electronic supplementary material, note S1, figure S1). We extracted, purified and dated collagen from four human teeth from Matenkupkum following routine protocols in the Oxford Radiocarbon Accelerator Unit [85]. Between 700 and 800 mg of dentine powder was extracted from each tooth using a dental drill equipped with a tungsten carbide drill bit. The samples underwent decalcification using 0.5 M HCl, washing with a 0.1 M NaOH solution and reacidification using 0.5 M HCl. Each step was interspersed with rinsing using distilled water. The extracted collagen was gelatinized using weakly acidic pH 3 water at 75°C in an incubator for 20 h. The gelatin was passed through an Ezee-filterTM to isolate insoluble residues and the supernatant was ultrafiltered using a Vivaspin 30 kD MWCO ultrafilter. The greater than 30 kD fraction was then collected, lyophilized and the dried product was weighed into tin capsules for combustion in a continuous flow mass spectrometer coupled with an elemental analyzer. A portion of the purified and analysed CO_2_ gas was collected, graphitized and pressed into targets for AMS measurement.

While few previous dates existed for Matenkupkum [86], Pamwak has already been extensively dated, with 59 radiocarbon dates already existing for the site [35] (electronic supplementary material, table S1). However, these dates have not been calibrated using the latest available calibration curves for the Pleistocene. Due to poor preservation, it was not possible to directly date the teeth themselves. As a consequence, it was necessary to try and build a comprehensive chrono-stratigraphic correlation for the human remains from this site. Following background correction, the radiocarbon dates are expressed as conventional radiocarbon ages (BP) and are shown in the electronic supplementary material, table S1. The dates are calibrated and converted into calendar ages (cal BP) using the most recent terrestrial calibration curves for the Northern Hemisphere [87] and the Southern Hemisphere [88], as shown in the electronic supplementary material, table S1. The comparison between the two datasets reveals that the differences are small given the uncertainties with regards to the effect of the variations in the Intertropical Convergence Zone and its impact on the radiocarbon dates from regions affected by it. For the calibration of marine shell dates, we used the Marine20 curve [89] and the local marine reservoir correction (DR) was calculated from the average of the five nearest locations to the respective site for which such values exist (for Pamwak this was DR = −96 ± 29 ^14^C yr).

Bayesian frameworks have been shown to greatly improve understanding of stratigraphic chronologies. Here, we use the OxCal platform [90] to combine the radiocarbon likelihoods with the relative stratigraphic information available for the sites of Matenkupkum and Pamwak. For Pamwak, the relationships between samples and units were included within the Bayesian model as prior information based on Fredericksen *et al.* [35]. The model consists of a series of phases, represented by the successive stratigraphic levels, and boundaries between them (see electronic supplementary material, note S1). Each phase in the model is separated by a phase boundary which represents the time required for the accumulation of each archaeological level. We used an outlier detection analysis [91] that probabilistically measures the degree to which determinations agree within the overall model structure and its components. We used a t-type outlier model and assigned 5% chances for each determination to be an outlier. In a first iteration (electronic supplementary material, figure S1), we built a Bayesian model containing all reliable radiocarbon determinations from Pamwak. Two determinations were excluded as they represented modern intrusions. In a second iteration, a further 10 radiocarbon dates were also excluded (electronic supplementary material, figure S2) and the results were compared.

### (b) Stable isotope analysis

We sampled eight human teeth available from Units C (*n* = 2) and D (*N* = 6) of Pamwak, constrained by date ranges of 7000–10 700 BP for Unit C and 10 800–14 100 cal BP for D (at 95.4%), as well as 75 teeth of terrestrial and marine fauna from Units A to E (see [35] and electronic supplementary material, note S1 for stratigraphic information) for stable isotope analysis. We also sampled four human teeth from Matenkupkum Square B as well as two samples of fauna excavated from Squares E, G, H, J, L. The latter dated to the late Holocene based on associated charcoal ages and the presence of glass, metal and other recent debris [9]. The human samples from Square B, however, while considered to be potentially Pleistocene in age, had not previously been dated [36], hence the human teeth underwent radiocarbon dating as well. We sampled one human tooth from the site of Sasi (Baun) on Lou Island in Manus (Square D), as well as 17 terrestrial and marine fauna from Squares A–G, all considered to be dated to approximately 2100 years ago to provide a more recent comparison for Pamwak within the same island group in Near Oceania. Finally, from the Teouma site in Vanuatu, we sampled 12 humans from Phase B, as well as 54 terrestrial and marine fauna. Mammal identifications were facilitated through comparisons with specimens from the ANU Archaeology and Natural History Osteology Laboratory reference collection. Fish identifications were undertaken using specimens from both the ANU Archaeology and Natural History Osteology Laboratory and the reference collection housed at the UMR 7209 of the National Museum of Natural History in Paris. The sample set (electronic supplementary material, tables S2 and S3) thus covers the earliest postulated human fossils in Oceania beyond the Sahul landmass and extends to the first occupation of Vanuatu by Lapita-affiliated populations and to post-Lapita contexts on Manus and New Ireland, enabling investigation of multiple phases of human adaptation to insular settings in the Pacific. More background information on the archaeological contexts can be found in the electronic supplementary material, note S1.

All sampled teeth were cleaned using air abrasion to remove any adhering external material. Enamel powder was obtained using gentle abrasion with a diamond-tipped drill along the full length of the buccal surface in order to ensure a representative measurement for the entire period of enamel formation. All enamel powder was pretreated to remove organics or secondary carbonate contaminants following existing protocols [23]. A subsection of analysed samples was checked for preservation using Fourier transform infrared spectroscopy (FTIR) as per Roberts *et al*. [23,40] (electronic supplementary material, note S2, table S4). Samples were progressively washed in 1% sodium hypochlorite for 60 min, rinsed and centrifuged three times in purified H_2_O, submerged in 0.1 M acetic acid for 10 min, and again triple rinsed and centrifuged in purified H_2_O. Samples were then frozen and lyophilized for 4 h and weighed into borosilicate vials. The vials were flush-filled with helium at 100 ml min^−1^ for 10 min. After the addition of 20 µl of 100% phosphoric acid, gases evolved from the samples were measured by stable carbon and oxygen isotope analysis using a Thermo Gas Bench 2 connected to a Thermo Delta V Advantage Mass Spectrometer at the Max Planck Institute for the Science of Human History, Jena, Germany. δ^13^C and δ^18^O values were compared against International Standards (IAEA-603 (δ^13^C = 2.5‰; δ^18^O = −2.4‰); IAEA-CO-8 (δ^13^C = −5.8‰; δ^18^O = −22.7‰); USGS44 (δ^13^C = −42.2‰)) and an in-house standard (MERCK (δ^13^C = −41.3‰; δ^18^O = −14.4‰)). Replicate analysis of MERCK standards suggests that measurement error is *ca* ± 0.1‰ for δ^13^C and ± 0.2‰ for δ^18^O. Measurement precision was studied through the measurement of repeat extracts from a bovid enamel standard (*n* = 20, ± 0.2‰ for δ^13^C and ± 0.3‰ for δ^18^O).

Following a Shapiro–Wilk test for normality and assessment of a histogram, a Mann–Whitney U test was used to determine whether there was a significant difference between the δ^13^C and δ^18^O of terrestrial and marine fauna analysed across the sites. Linear regressions were performed on the combined marine and terrestrial faunal datasets for Pamwak, Sasi and Teouma, while a separate linear regression was performed for just the terrestrial fauna at Teouma. Kruskal–Wallis tests, followed by pairwise Wilcox tests, were also performed on the terrestrial faunal dataset from Pamwak to explore whether δ^13^C and δ^18^O varied through time (i.e. by phase). All statistical analyses were conducted using the free program R software [92].

### (c) Phytolith analysis of dental calculus

We sampled dental calculus from five human teeth from Matenkupkum Square B and analysed the extracted microparticles (as per [30]). Detailed information on the teeth sampled and methods used can be found in the electronic supplementary material, text S3. The dental calculus samples were taken from what was interpreted to be Burials 1 (2 MATB1 2/3), 2 (8 MATB2 5/27), 3 (61 MATB3 31/35), 4 (66 MATB4 1/5) and 5 (73 MATB5 3/89) (electronic supplementary material, notes S1 and S3). Meanwhile the dates and SI results come from Burials 1 (1 MATB1 1/3) and 2 (8 MATB2 5/27, 9 MATB2 6/27, 12 MATB2 9/27). As a result, there is one tooth that had both calculus and was dated; the other teeth that were dated are associated with burials that had a tooth analysed for calculus.

## 4. Results

### (a) Radiocarbon dating and Bayesian modelling

**Figure 2.**
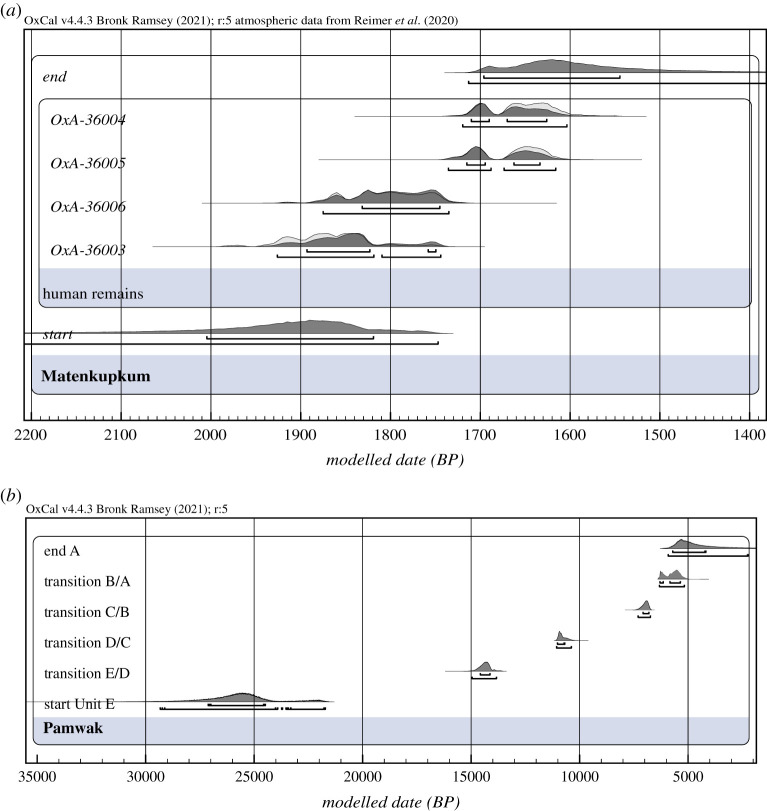
Modelled chronometric data for (*a*) the directly dated human teeth from Matenkupkum and (*b*) start boundaries for Pamwak representing the unit transitions produced by the first Bayesian model (see electronic supplementary material, Note S1).

Our dates from the four human teeth from Matenkupkum address a stark lack of direct dates on human fossils from the Bismarck Archipelago, with the only others being available from the Lapita site at Reber-Rakival on Watom Island, off the coast of East New Britain [93,94]. The collagen yields ranged between 1 and 3% and all C/N ratios fell well within normal values (3.2: normal range 3.2–3.6). The results of these dates are shown in the electronic supplementary material, table S5. In order to place the new and old measurements in a comparable statistical framework, we attempted to build Bayesian models for the two dated sites. In the case of Matenkupkum, we used a simple, single-phase Bayesian model to calculate the most likely start and end dates for the deposition of the teeth at the site, using both Northern and Southern Hemisphere calibration curves for comparison. As shown in the electronic supplementary material, table S6, the start and end phase boundaries are statistically indistinguishable and differ by 10 to 90 years, giving an age range of between 1870 and 1630 or 2090 and 1430 cal BP (68.3/95.4%) with a mean of 1750 ± 150 cal BP for the deposition of the teeth, a date much younger than had been assumed by the excavation team [36], highlighting that the shallow deposits at the rear of the cave cannot be simply correlated to the previously excavated Pleistocene sequence (figure 2).

**Figure 3.**
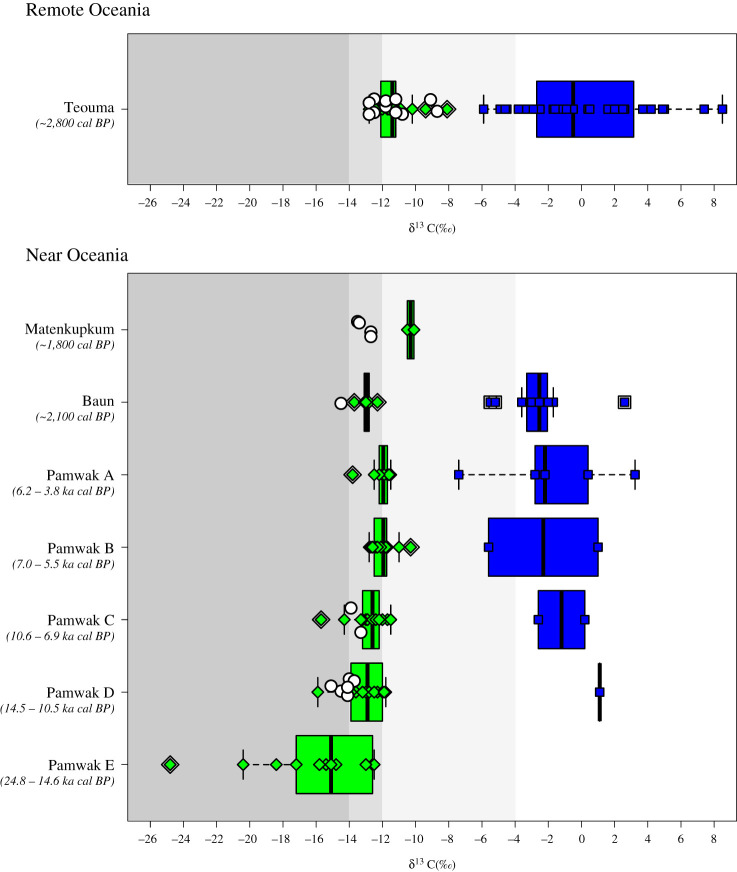
Stable carbon isotope (δ^13^C) measurements on humans (white circles), terrestrial fauna (green diamonds) and marine fauna (blue squares) from the sampled contexts of Baun (Manus Island), Matenkupkum (New Ireland), Teouma (Vanuatu) and Pamwak (Manus Island). The boxes of the boxplots show the median and the lower (25%) and upper (75%) quartiles. The whiskers (connected to the boxes by a dashed line) encompass all data points within 1.5x the interquartile range of the box. All points that fall beyond this range are shown as ‘outliers’ bordered by white and black lines though they have not been removed from any of the statistical analyses. The shading (increasingly light grey shading from dark grey to white from left to right) depicts the different approximate expected ranges for dense canopy forest feeding (less than −14‰), 100% C_3_ consumption (−14 to −12‰), mixed C_3_/C_4_ consumption (−12 to −4‰) and 100% C_4_/marine consumption (greater than −4‰) discussed in the main text and the literature and references cited therein.

For Pamwak, the first model (Bayesian 1) incorporated 57 dates previously obtained from three different laboratories and using charcoal and *Celtis* sp. seeds, as well as marine shell (electronic supplementary material, table S1). We use five phases to represent the five Units identified at the site (A–E) and the ages were nested within them (electronic supplementary material, figure S2). In the second iteration (Bayesian 2) (electronic supplementary material, figure S3), the ten ages identified in Bayesian 1 as complete outliers were excluded (OZC-835, ANU-7124, ANU-6925, ANU-7508, ANU-7509, OZD-781A, Wk-7674, ANU-8252, OZD-779, ANU-6978) and the results were compared (electronic supplementary material, figure S2). The differences in the start boundaries between the two models were minimal which suggest that the ages assigned to each unit are relatively robust. Using a Date function, we also tried to calculate the most likely duration of each Unit, the results of which are used in figure 3 (electronic supplementary material, table S1). We use the results from Bayesian 1 (all dates included) (electronic supplementary material, figure S1) in our discussion below (electronic supplementary material, table S7). Due to the very large errors associated with some of the earlier determinations, the start of Unit E is calculated very broadly to between 27 and 21 ka cal BP and its duration between 24.8 and 14.6 ka cal BP, with the subsequent transitions and spans dated as follows: the start of Unit D is placed approximately 15–13.5 ka cal BP with duration between 14.5–10.5 ka cal BP, the start of Unit C between 11.0–9.8 ka cal BP and its duration at 10.6–6.9 ka cal BP, and the start of Unit B at 7.3–6.7 ka and its duration between 7.0–5.5 ka cal BP, whereas Unit A starts at 6.3–5.1 ka cal BP with an estimated duration between 6.2–3.8 ka cal BP (all ranges in 95.4%) (figure 2).

The Pamwak human teeth are constrained by dates on material above and below from direct or equivalent stratigraphic contexts on charcoal and *Celtis* sp. seeds. In order to calculate, in a probabilistic way, the age of the different teeth in the sequence, we also built an additional Bayesian framework in which we incorporated the chrono-stratigraphic information pertinent to each human sample only (electronic supplementary material, figure S4). Based on the currently available data, the earliest human tooth (PAMH7) in the site comes from Unit D and is constrained by a date above it (ANU-8251) and a date stratigraphically below it in a neighbouring quadrant (ANU-8243). The Bayesian age estimate for PAMH7 was calculated at 12 260–11 420 or 12 510–10 940 cal BP at 68.3/95.4%, with a mean age of 11 770 ± 410 cal BP. PAMH5 is constrained by dates of 12 620–11 940 BP (ANU-8243) below and 11 390–10 760 BP (OZD-779) above in a neighbouring square and appears to be as old (12 100–11 240 or 12 460–10 990 cal BP at 68.3/95.4%), with a mean age of 11 700 ± 390 cal BP that is statistically indistinguishable to PAMH7. The youngest tooth (PAMH2) from Unit C is constrained by dates stratigraphically equivalent to it (ANU-7122) and (ANU-8239) from the same 5 cm spit, and a date stratigraphically some way below it (ANU-8240), all from a neighbouring quadrant. From the same quadrant as the tooth, and below it, comes a date of 11 840–10 710 BP cal BP (Wk-7670). However, given that PAMH2, PAMH3 and PAMH4 belong to the same individual, and with the last two falling to between 10 080 ± 620 cal BP and 10 330 ± 360 cal BP (mean modelled age estimates), we may assume that the individual all three teeth belonged to was deposited at the site at around 10 100 cal BP (electronic supplementary material, figure S4 and note S1).

The teeth are all part of a secondary burial assemblage seemingly cached on ledges above the shelter floor which entered the deposit by being progressively displaced from above. Nearly all the associated bones, however, are found in Units C and D, as were the teeth, with none below D and only small amounts in B and a single bone in Unit A. This implies a different situation to Matenkupkum, given that we have a series of tightly constrained and consistent dates for excavation spits containing the human remains. Moreover, while the Matenkupkum teeth were found in relatively shallow deposits at the back of the cave (electronic supplementary material, note S1), as noted above, the Pamwak human remains are firmly associated with Units C and D and must have been deposited in these layers at least prior to their respective covering by the overlying unit. Nevertheless, although the constrained Bayesian model focused on the human remains provides a potentially more high-resolution window into the ages of human remains in the Pamwak sequence, in the absence of direct dates the broader ages for the units remain the most conservative ages for the human remains and are what we use in figure 3 and as the primary reference for the Discussion below. For further details of the dating of the Pamwak teeth as well as for the full Bayesian models, the reader is referred to the electronic supplementary material, note S1, figures S2–S4.

### (b) Stable isotope analysis

The results of the δ^13^C analysis of terrestrial fauna, marine fauna and human teeth from the sites of Pamwak, Sasi, Matenkupkum and Teouma are shown in figure 3. More detailed plots of the δ^13^C and δ^18^O results for each site and, in the case of Pamwak, unit/phase, are provided in the electronic supplementary material, tables S2 and S3, and figures S5–S8. No evidence for secondary carbonate at 710 cm^−1^ (e.g. calcite) was observed for any of the FTIR sample spectra (electronic supplementary material, note S2, figures S9 and S10, and tables S4 and S8–13) For each site, distinct and non-overlapping δ^13^C values are demonstrated for terrestrial and marine fauna, as has been shown elsewhere in the Pacific (e.g. [23]). This is confirmed by a Mann–Whitney U test which demonstrates a significant δ^13^C difference between marine and terrestrial fauna at all sites (*W* = 4800, *p* < 0.05). These results suggest that (i) the sampled terrestrial fauna at the sites did not have access to C_4_ resources and (ii) that human reliance on terrestrial versus marine resources can be relatively confidently determined. The human δ^13^C datapoints from Pamwak Units C and D provide an insight into the diet of some of the earliest preserved human individuals found eastwards of the Sahul landmass on Manus Island, indirectly dated here to approximately 12 000–10 000 cal BP. δ^13^C values of five individuals between −15.1 and −14.0‰ are indicative of a full reliance on C_3_ terrestrial resources and, particularly, those influenced by a closed forest canopy (see [39]). The remaining three values between −13.9 and −13.3‰ include both individuals from the younger Unit C and an infant from near the top of Unit D. They show a reliance on C_3_ terrestrial resources, likely still from forest/woodland biomes.

Our dating of the Matenkupkum individuals to around 1750 ± 150 cal BP places them within a post-Lapita timeframe. Values between −13.5 and −12.7‰ for the four individuals sampled at this site, as in the case of Pamwak, suggest terrestrial foraging, likely with a strong component of forest and woodland resources on New Ireland at this time, perhaps alongside the use of more open mosaic terrestrial habitats. Sasi is identified as a post-Lapita context dating to 2100 years ago [41,95]. Here, the sampled individual has a δ^13^C value of −14.5‰ indicating a clear reliance on tropical forest resources. Our data from the Lapita context of Teouma, Vanuatu add to the existing bone collagen data from the earliest contexts of human habitation of Remote Oceania. Our human values from this site, ranging between −12.8 and −8.7‰ likely suggest the inclusion of more marine resources in human diets than in the other contexts analysed. It is important to note that the terrestrial faunal δ^13^C baseline at Teouma is higher than those observed at the other sites (electronic supplementary material, figures S5–S7). This could suggest the availability of more mosaic terrestrial environments, or the mixed exploitation of closed tropical forests alongside C_3_-domesticated plants, domesticated animals (e.g. pigs), and local plants and animals (e.g. bats and tortoises—[31,70]) living in more open patches. Although they are notoriously difficult to interpret, particularly for humans, it is possible that our δ^18^O data may help to differentiate between these varying scenarios.

A Mann–Whitney U test demonstrated a significant δ^18^O difference between the sampled marine and terrestrial fauna (*W* = 4447, *p* < 0.05), suggesting that δ^18^O may indeed be useful for differentiating terrestrial and aquatic ecologies as a result of different body water sources and physiologies [82]. Linear regression analyses of δ^18^O and δ^13^C of marine and terrestrial fauna together showed small, positive correlations at Pamwak (adjusted *R*^2^ = 0.51, *p* = <0.05), Sasi (adjusted *R*^2^ = 0.24, *p* = <0.05) and Teouma (adjusted *R*^2^ = 0.37, *p* = <0.05), further indicating that both isotopic parameters differentiate between marine and terrestrial habitats. At Pamwak, the stronger correlation may be driven by two murids with very low δ^13^C and δ^18^O (electronic supplementary material, table S2). Similarly, low values were also noted for rodents in Late Pleistocene Sri Lanka [39,40] and may indicate feeding on plants growing in dark, cave conditions. In Vanuatu, a stronger correlation exists when both marine and terrestrial fauna are considered, than when just terrestrial fauna are considered (adjusted *R*^2^ = 0.15, *p* = <0.05). This may support the argument that higher human δ^13^C is a result of increased marine reliance rather than use of more open environments. Nevertheless, similar work on terrestrial and marine fauna in Wallacea failed to show a δ^18^O correlation with δ^13^C [23] and the correlations shown here remain weak, urging caution in the use of δ^18^O as a habitat indicator. A comparison of terrestrial faunal δ^18^O and δ^13^C over time at Pamwak, however, does provide further useful insights into changing environmental conditions. A Kruskal–Wallis test followed by Wilcox pairwise comparisons indicate statistically significant differences between phases in both cases (δ^13^C: *X*^2^ = 23.08, d.f. = 4, *p* < 0.05; δ^18^O: *X*^2^ = 20.02, d.f. = 4, *p* < 0.05), with Unit E being different from Units A and B in both cases (less than 0.05) (electronic supplementary material, tables S14 and S15), suggesting an opening of forests through time.

### (c) Phytolith analysis of dental calculus

Very few microparticles were recovered. However, there is evidence for the use of woody shrub or tree leaves, consumption of unknown starchy plants, and obsidian use or manufacture within the dental calculus from Matenkupkum (electronic supplementary material, note S3, figure S11, and tables S16 and S17). This site has evidence of obsidian originating from New Britain by at least 12 000 years ago [9]. Dental calculus has the potential to incorporate anything that comes into contact with the mouth, which can include dust and debris from manufacturing [96]. It is quite likely that this obsidian was either inhaled during manufacture of a tool or chipped off while a tool was held in the person's mouth.

## 5. Discussion and conclusion

Our new radiocarbon age model for Pamwak indicates occupation of this site spanned between 26 000 years ago (if OZC-835 can be trusted) or 22 000 years ago and the recent past. This adds further detail to our understanding of the process of human dispersal into island settings beyond the Sahul landmass during the Late Pleistocene. The earliest evidence for human arrival in the Bismarck Archipelago remains that from the sites of Buang Merabak (44–40 000 years ago) and Matenkupkum (40–39 000 years ago) and others on New Ireland [44], and Kupona Ni Dari, with optically stimulated luminescence dates starting at 39 800 BP [46], and Yombon on New Britain [45]. Our new dating for Pamwak, as well as the fact that deeper undated cultural layers exist at the site, similarly supports a relatively early human arrival on Manus Island [45]. Our chronological information for the human remains found from Units D and C (approx. 14 500–6 900 cal BP) from Pamwak, and the direct dating of human teeth found from expected Pleistocene layers at Matenkupkum (yielding a late Holocene date), however, further emphasize the large gap that exists between the first arrival of humans in this part of Oceania, and the preserved human fossil record. In the case of Matenkupkum, the late ages for the human teeth found during the later excavations of Square B [36] fit with the much shallower nature of this deposit relative to the earlier main excavation towards the front of the cave and suggest that Square B cannot be simply correlated with the Pleistocene record of the cave without a more rigorous dating programme. The chronological distance between human colonization and the preserved fossil record is problematic given a growing focus on potential admixture between arriving *H. sapiens* and resident Denisovan populations in the region during the Late Pleistocene that have left their mark on the genetic make-up of modern Oceanian populations [14]. It also makes it difficult to determine the exact dietary reliance on forest or marine resources of the first human populations stepping out into the island chain of the Bismarck Archipelago.

Nevertheless, we provide the earliest direct information relating to the diets of humans moving out into the Near Oceanic parts of Oceania beyond Sahul. Our comprehensive baseline of terrestrial fauna and marine fauna from the sites sampled confirm the efficacy of this approach for discerning human use of tropical interior forest resources and oceanic habitats [23]. The earliest human samples come from Units C and D of Pamwak, the oldest of which (PAMH7) was indirectly dated to as early as approximately 11 800 years ago. The stable carbon isotope values of these individuals suggest a focus on interior island resources. An isotopic study on Timor-Leste and Alor found that the earliest humans in southern Wallacea likely practiced a specialized marine adaptation approximately 36 000 years before shifting to an increasing reliance on interior tropical forests from around 12 000 years ago [23]. Given the chronological limitations noted above, we cannot currently rule out the importance of coastal adaptations to the earliest human arrivals in the Bismarck Archipelago. It remains possible that marine resources, observed at Matenkupkum and other early coastal sites, played an important role in earliest colonization, as has been observed for Timor-Leste [23]. Such resources likely continued to be exploited into the terminal Pleistocene. However, our isotopic data from Pamwak confirm the development of a specialized adaptation to insular tropical forests by the end of the Pleistocene in island Oceania (cf. [9]). This fits with increasing zooarchaeological evidence for the dominant use of native forest birds, bats, rodents and reptiles, and botanical evidence for the use of various plants in the Bismarck and Solomon Archipelagoes at this time [45,49]. Observations of the transport of small mammals to new islands approximately 20 000 years ago [86], alongside recent evidence for active use of fire in forest management on Manus by 5000 cal BP [97], potential arboriculture even earlier [63], and our δ^13^C and δ^18^O faunal isotopic evidence for an opening of forest between Pamwak Units E and A (figure 3), may suggest these Pleistocene tropical adaptations involved active human management of resources. Alongside documented Late Pleistocene forest management through burning on the New Guinea mainland [3,98], and perhaps also in northeastern Australia [99], this evidence from insular Oceania potentially adds to a growing recognition of deep time tropical forest shaping by humans in the Pleistocene—although more detailed palaeoecological research is required to demonstrate this definitively for the Bismarck Archipelago.

Methodological issues and preservation challenges will need to be overcome to investigate this further for the earlier period (see also [22,23,62]). However, our stable isotope and microparticle data also contribute to an increasing emphasis on the role of agroforestry during later periods of colonization of Remote Oceania [29,30,71]. The expansion of Lapita populations along the coasts of Near Oceania and out into the Pacific during the late Holocene, as well as subsequent dispersals in this part of the world, have rightly been associated with impressive oceanic navigation skills and marine adaptations [100–103]. Furthermore, these human movements are linked to the arrival of domesticates (e.g. pigs, chickens) and commensals (e.g. rats) into various island ecosystems, with corresponding consequences for local biodiversity and soil erosion [26,47,104]. The early and persistent management of endemic tropical forest plants and forest cover during this early phase of settlement has, however, perhaps been underestimated. Our stable isotope data from Lapita contexts at Teouma, Vanuatu suggest that the earliest human arrivals in this part of the world were already incorporating significant tropical plant carbohydrates into their diets, also suggested by a recent study of dental calculus [30]. As has been shown by comprehensive work on the bone collagen of individuals from the same site, this was likely undertaken alongside the incorporation of marine resources, with C_3_ domesticate crops and domesticated animals also being added over time [24,38]. On the other hand, our data from the late Holocene samples from Matenkupkum and Sasi suggest a similar, ongoing focus on tropical forest (e.g. native bats) and domesticated terrestrial resources, although without clear evidence for marine protein reliance. Overall, this adds to growing recognition that human dispersals and adaptations in Oceania between 4000 and 1700 years ago were characterized by variable, locally framed adaptations, depending on island size and type, available resources, population mobility, and local ecology and geology [26,28], with tropical forests likely being used and managed alongside introduced domesticates and marine resources.

Additional work is needed to explore the chronology and ecological context of human arrival into insular Oceania beyond Sahul during the Late Pleistocene and early Holocene. Our study further highlights the lack of early human fossils in the region, with new dating efforts confirming a late chronology for human remains at the sites of Pamwak and particularly Matenkupkum. The application of Zooarchaeology by Mass Spectrometry (ZooMS) holds much promise in this regard (for an overview see [105]) and has shown its capacity to identify hominin remains from previously unidentifiable fragmented faunal datasets and has already been shown to be viable in the tropics, including in the Pacific region [106]. Nevertheless, our isotopic data add to an emerging understanding of how early human arrivals might have adapted to this part of the world. The tropical islands of the Pacific have often been portrayed as challenging prospects for human colonization (e.g. [107,108]), requiring maritime navigation and sophisticated adaptations to depauperate tropical forest settings. However, zooarchaeological, archaeobotanical, palaeoecological, chronological and now isotopic insights from insular Oceania highlight that our species had moved into the islands beyond Sahul and developed sophisticated adaptations to their forests, as early as the Late Pleistocene. By the terminal Pleistocene, humans were even actively managing these insular tropical habitats, with evidence for the translocation of tropical animals, plants and human-caused burning out beyond Sahul as early as approximately 20 000–12 000 years ago [9], perhaps suggesting that the concept of ‘transported landscapes’ used to describe the later movements of the Lapita Cultural Complex through the Pacific [109] also has a pre-Holocene relevance. Certainly, it is becoming increasingly apparent that the varied tropical forests of Oceania were as significant in both Late Pleistocene and Holocene human colonization and long-term settlement of island ecosystems as the coasts and marine settings that enabled humans to move into these new Pacific contexts.

## Data Availability

All sampled specimens are stored at the Australian National University, the University of Otago, and the Vanuatu Cultural Centre for curation. All of the data reported in this manuscript are provided within the main text, figures or electronic supplementary material [110].
